# Influence of TNFα–308 and T676G TNF-RII polymorphism on response to etanercept and posibility to discontinue tretment

**DOI:** 10.1186/1546-0096-9-S1-P277

**Published:** 2011-09-14

**Authors:** J Vojinovic, G Susic, D Lazarevic, J Basic, I Nikolic, N Damjanov

**Affiliations:** 1 Department of Pediatric Rheumatology, University Clinical Center Nis, Serbia; 2 Institute of Rheumatology Belgrade, Faculty of Medicine Nis, Serbia; 3 Institute of Biochemestry, Faculty of Medicine Nis, Serbia

## Background

Genetic contribution of TNFὰ–308 promoter and T676G TNF-RII polymorphism on response to TNF-blocking agents in JIA is not yet well established.

## Methods

Genomic DNA was extracted and TNFὰ–308 promoter and T676G TNF-RII polymorphism was evaluated using the PCR-RFLP method in 60 JIA patients treated with etanercept. Time cut of point for outcome data analysis was 4 years.

## Results

Average duration of etanercept therapy was 34.61±12.11 months. Disease subtype distribution was 6.78% systemic, 54.24% poly RF- and extended-oligo, 18.64% poly RF+, 16.95% ERA and 3.38 PsA. The distribution of TNFὰ308 and T676G genotypes was not significantly different among JIA subtypes. TNFὰ308 genotypes distribution was 6.78% AA, 30.51% GG and 62.71% GA while T676G genotypes were 59.3% TT, 8.3% GG and 26.4% TG. T676G genotype polymorphism did not significantly influenced outcome. ACR Pedi 30,50,70 and 100 improvement was significantly faster and sustained in TNFὰ308 GG-genotype patients compared to GA genotype (results shown in table:*significant compared to 1 year; a-significant compared to GG). Treatment induced remission in 35.14%, had to be reintroduced due to disease worsening in 16.22%, disease was in remission under medication in 21.62% or still active in 24.32% GA patients and in 38.9%, 16.7%, 27.8% and 11.1% in GG patients, respectively. Patients with systemic or RF+ disease course were mostly treatment resistant in both genotypes. (Table 1)

**Table 1:** 

ACR	30		50		70		100	
%	GG	GA	GG	GA	GG	GA	GG	GA

1 year	5.6	18.9^a^	22.2	35.14^a^	22.2	32.43^a^	50.9	13.51^a^

2 years		14.71^a^	5.6*	17.65*^a^	44.4*	44.12*	50.0	23.52*^a^

## Conclusion

JIA patients with GG TNFὰ308 genotype can achieve better outcome and etanercept treatment can be stopped earlier compared to GA genotype patients who need two years of treatment to achieve same results. TNFὰ308 genotype could be useful clinical predictive biomarker for treatment response in all disease subtypes except systemic and RF positive JIA.

